# Novel Coagulation Factor VIII Gene Therapy in a Mouse Model of Hemophilia A by Lipid-Coated Fe_3_O_4_ Nanoparticles

**DOI:** 10.3390/biomedicines9091116

**Published:** 2021-08-30

**Authors:** Yung-Tsung Kao, Yen-Ting Chen, Hueng-Chuen Fan, Tung-Chou Tsai, Shin-Nan Cheng, Ping-Shan Lai, Jen-Kun Chen, Chuan-Mu Chen

**Affiliations:** 1 Department of Pediatrics, Tungs’ Taichung Metroharbor Hospital, Taichung 435, Taiwan; g106052319@mail.nchu.edu.tw (Y.-T.K.); fanhuengchuen@yahoo.com.tw (H.-C.F.); t12241@ms.sltung.com.tw (S.-N.C.); 2 Department of Life Sciences, Ph.D. Program in Translational Medicine, National Chung Hsing University, Taichung 402, Taiwan; abelchen03@gmail.com (Y.-T.C.); tctsai86@gmail.com (T.-C.T.); 3 Ph.D. Program in Tissue Engineering and Regenerative Medicine, National Health Research Institutes and National Chung Hsing University, Taichung 402, Taiwan; jkchen@nhri.edu.tw; 4 Department of Rehabilitation, Jen-Teh Junior College of Medicine, Miaoli 356, Taiwan; 5 Hemophilia Care and Research Center, Tri-Service General Hospital, Taipei 114, Taiwan; 6 National Defense Medical Center, Department of Pediatrics, Taipei 114, Taiwan; 7 Department of Chemistry, National Chung Hsing University, Taichung 402, Taiwan; pslai@dragon.nchu.edu.tw; 8 Institute of Biomedical Engineering and Nanomedicine, National Health Research Institutes, Miaoli 350, Taiwan; 9 The iEGG and Animal Biotechnology Center, National Chung Hsing University, Taichung 402, Taiwan; 10 Rong Hsing Research Center for Translational Medicine, Taichung Veterans General Hospital, Taichung 407, Taiwan

**Keywords:** hemophilia A mouse, gene therapy, DPPC-Fe_3_O_4_, nanoparticle, coagulation FVIII

## Abstract

Hemophilia A is a bleeding disease caused by loss of coagulation factor VIII (FVIII) function. Although prophylactic FVIII infusion prevents abnormal bleeding, disability and joint damage in hemophilia patients are common. The cost of treatment is among the highest for a single disease, and the adverse effects of repeated infusion are still an issue that has not been addressed. In this study, we established a nonviral gene therapy strategy to treat *FVIII* knockout (*FVIII* KO) mice. A novel gene therapy approach was developed using dipalmitoylphosphatidylcholine formulated with iron oxide (DPPC-Fe_3_O_4_) to carry the B-domain-deleted (BDD)-*FVIII* plasmid, which was delivered into the *FVIII* KO mice via tail vein injection. Here, a liver-specific albumin promoter-driven BDD-*FVIII* plasmid was constructed, and the binding ability of circular DNA was confirmed to be more stable than that of linear DNA when combined with DPPC-Fe_3_O_4_ nanoparticles. The *FVIII* KO mice that received the DPPC-Fe_3_O_4_ plasmid complex were assessed by staining the ferric ion of DPPC-Fe_3_O_4_ nanoparticles with Prussian blue in liver tissue. The bleeding of the *FVIII* KO mice was improved in a few weeks, as shown by assessing the activated partial thromboplastin time (aPTT). Furthermore, no liver toxicity, thromboses, deaths, or persistent changes after nonviral gene therapy were found, as shown by serum liver indices and histopathology. The results suggest that this novel gene therapy can successfully improve hemostasis disorder in *FVIII* KO mice and might be a promising approach to treating hemophilia A patients in clinical settings.

## 1. Introduction

Hemophilia is a genetic disorder caused by the loss of coagulation factor genes. Approximately 30% of hemophilia patients have spontaneous mutations, and others have a family genetic history [1,2]. Clinically, hemophilia is diagnosed if the coagulation factor activity in plasma is less than 40% [3]. Patients with severe hemophilia have less than 1% of normal coagulation activity, and recurrent spontaneous bleeding episodes from subcutaneous tissues and joints will cause hematomas [4,5]. Hemophilia is divided into different types according to the lack of different coagulation factors; among them, approximately 70–80% of cases are hemophilia A. Hemophilia A is a recessive X-linked hereditary disease, and the most common mutation is the intron 22 inversion of the *FVIII* gene [6,7]. As a result, the function of this gene is lost, affecting subsequent responses to the intrinsic coagulation pathway and causing coagulopathy [8]. In the clinic, almost all patients need long-term supplementation with FVIII to maintain the activity of a particular coagulation factor. However, because treatment with FVIII, either purified from plasma or produced from cultured cells, is expensive, most patients do not acquire proper medical care [9,10].

Recently, the new medicines for treating hemophilia A have been developed. Emicizumab is a bispecific antibody which can connect the activated factor IX and factor X. It can activate the factor X and recover the clotting problem of hemophilia A [11,12]. Bypassing agents, such as FEIBA and NovoSeven, provided alternative treatments for hemophilia that are supplemented by a key clotting factor to complete the coagulation pathway. The FEIBA supplies several clotting factors to form the thrombin and improve the coagulation activity in hemophilia patients. In contrast, the NovoSeven is a recombinant activated factor VII which can enhance the formation of fibrin in hemophilia patients [13]. Rebalancing agents provide another therapeutic strategy that can restore the coagulation ability by inhibiting the anticoagulants, such as activated protein C (APC), antithrombin (AT), and tissue factor pathway inhibitor (TFPI). Several rebalancing agents have been developed and demonstrated their efficacy in hemophilia A animal experiment and hemophilia patients in clinical trial. It might be a potential candidate to treat hemophilia A in the future [14].

Previous studies have indicated that the expression level of B-domain-deleted (BDD)-FVIII is seventeen times higher than that of full-length FVIII in cultured cells and in the milk of transgenic animals [15,16]. Moreover, there is no obvious difference in the FVIII activity between BDD-FVIII and full-length FVIII, and BDD-FVIII can even maintain long-term coagulation function [17,18]. Currently, plasma-purified native FVIII or recombinant human BDD-FVIII protein is the principal treatment for hemophilia A. The recombinant human FVIII protein is produced by CHO cells using serum-free culture medium, which reduces the risks of blood-sourced pathogens [19,20]. However, the half-life of recombinant human FVIII is relatively short. Patients with severe disease need to be injected several times a week. Furthermore, approximately 10% to 40% of hemophilia A patients develop inhibitory antibodies and show diminished treatment effects [21,22]. Based on these adverse effects, new gene therapy or gene editing approaches have been developed to improve current treatments.

Viral vectors or nonviral vectors are two strategies to conduct gene therapy. The most popular virus-mediated gene therapy is a lentivirus system that can be used as a DNA vector, bringing foreign genes into cells to achieve gene therapy. However, the immune system will produce inhibitory antibodies if the target gene transduces antigen-presenting cells. Hence, this treatment will suppress coagulation factor function and fail to achieve long-term therapeutic efficacy [23]. In addition, lentivirus vector-delivered foreign genes will be randomly inserted into the host’s genome, damaging normal gene loci or activating oncogenes and causing other diseases [24]. In contrast, adeno-associated virus (AAV) vector-based gene therapy showed promise in terms of both safety and delivery efficiency in liver diseases [25]. A first-in-human clinical trial of an AAV5-*hFVIII*-SQ vector for treating 15 adults with severe hemophilia A concluded that the therapy was beneficial and safe [26]. However, the foreign gene size is limited in the AAV vector, and some patients will generate neutralizing antibodies or endoplasmic reticulum stress to influence the treatment effect [27].

Dipalmitoylphosphatidylcholine (DPPC), a lipid-coated nanoparticle, has been developed as a nonviral delivery system to transport DNA or drugs into cells. The structure of DPPC is similar to that of the cell membrane, which is amphiphilic, and its amino group at the hydrophilic end contains a positive charge, which can interact with negatively charged DNA [28]. Furthermore, DPPC has low toxicity and high biocompatibility, which is why it can be efficiently transported into the cell by endocytosis [29]. Superparamagnetic nanoparticles have been used in biomedical applications, such as drug delivery, cell tracking, and magnetic resonance imaging (MRI) [30,31]. Among all superparamagnetic nanoparticles, iron oxide (Fe_3_O_4_) nanoparticles are most widely used for their biosafety [32,33] and are mainly distributed in the liver and spleen organs [34].

In this study, we aimed to develop novel DPPC-Fe_3_O_4_ nanoparticles carrying a liver-specific BDD-*FVIII* expression plasmid as a nonviral gene delivery system for hemophilia A treatment in an *FVIII* knockout mouse model. We hypothesize that after combination with DNA, the DPPC-Fe_3_O_4_ nanoparticles will be able to change the configuration and form a more stable biomaterial that can easily enter liver cells. DPPC-Fe_3_O_4_ nanoparticles can also protect against DNA degradation in the blood circulation. The therapeutic efficacy and safety of DPPC-Fe_3_O_4_-driven gene therapy in a mouse model of hemophilia A were extensively elucidated.

## 2. Materials and Methods

### 2.1. Animals

The FVIII knockout mouse strain (B6;129S-*F8^tm1kaz^*/J) was purchased from the Jackson Laboratory (Bar Harbor, ME, USA) and the C57BL/6J control mouse strain was purchased from BioLASCO Taiwan Co., Ltd. (Taipei, Taiwan). All animals were kept in an IVC system at our animal facilities, and animal experimental procedures followed the Guide for the Care and Use of Laboratory of the National Institutes of Health. Moreover, all animal experiments were approved by the Institutional Animal Care and Use Committee of National Chung Hsing University (IACUC No. 104-120).

### 2.2. Liver-Specific Promoter Construction

For cloning of liver-specific promoters, the α1-antitrypsin promoter (Pα1-AT), α1-antitrypsin enhancer (Eα1-AT), α1-antitrypsin enhancer and promoter (EPα1-AT), and mouse albumin promoter (PmAlb) were amplified by PCR from mouse genomic DNA and inserted into the pGEM-T Easy vector (Promega, Madison, WI, USA). Primer sequences are listed in Table 1.

### 2.3. Dual Luciferase Assays

A dual luciferase assay system was used to analyze liver-specific promoter activities. Briefly, all promoters were cloned into the pGL3-enhancer vector containing a luciferase reporter gene. Seven cell lines, Hepa1-6, MEF, C2C12, CHO, A549, Caco-2, and Ca9-22, were used in this study. Cells were transfected with the liver-specific promoter-pGL3 plasmid by the Lipofectamine 2000 system (Invitrogen, Carlsbad, CA, USA). Transfected cells were lysed, and supernatants were collected for luminescence detection. First, 100 µL of LarII reagent (Promega) was mixed with a 20 µL of cell sample, and firefly luciferase was measured at O.D. 560 nm. Subsequently, 100 µL of Stop & Glo reagent (Promega) was added, and Renilla luciferase activity was measured at O.D. 560 nm. The promoter activity was calculated by the ratio of O.D. values of firefly luciferase and Renilla luciferase [35,36].

### 2.4. Gene Therapy Plasmid Construction and Production

**Figure 1:**
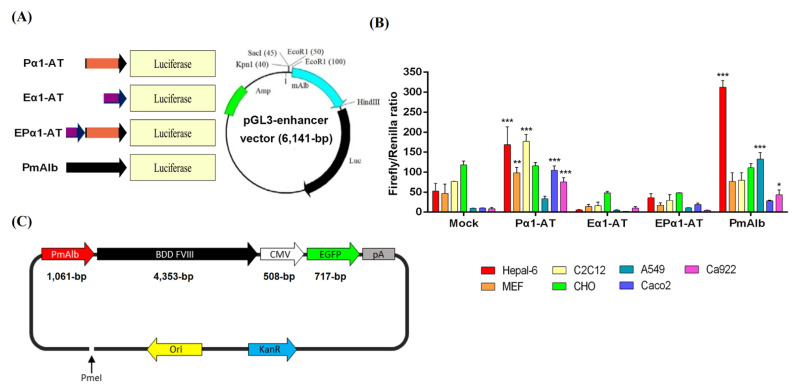
Liver-specific promoter construction and promoter activity measurement. (**A**) Four different liver-specific promoters, Pα1-AT, Eα1-AT, EPα1-AT, and PmAlb, were generated and inserted into the pGL3-enhancer vector using the luciferase gene as a reporter for the promoter assay. (**B**) Detection of the firefly/Renilla ratio of each promoter in seven different cell lines (Hepa1-6, MEF, C2C12, CHO, A549, Caco-2, and Ca922) by dual-luciferase assays. Mock group: transfected cells as a background control. (**C**) PmAlb-*BDD-FVIII*-pCMV-*EGFP* plasmid map. Data are presented as the mean ± SD (*n* = 3), * *p* < 0.05 vs. the mock group, ** *p* < 0.01 vs. the mock group, *** *p* < 0.001 vs. the mock group.

The mouse albumin promoter and B-domain-deleted (BDD) human *FVIII* cDNA were inserted into the pAdTrack vector (Addgene, Watertown, MA, USA) combined with pCMV-*EGFP* to form a binary expression cassette (Figure 1). The PmAlb-BDD-*FVIII*-pCMV-*EGFP* gene therapy plasmid was amplified in 500 mL of bacterial culture and purified using an EndoFree Plasmid Maxi Kit (Qiagen, Hilden, Germany).

### 2.5. Synthesis of DPPC-Fe_3_O_4_ Nanoparticles

The procedure to obtain oil phase Fe_3_O_4_ were as follows: First, 2 mmol of iron (III) acetylacetonate (Sigma-Aldrich, St. Louis, MO, USA) was added with 5 mmol of 1,2-hexadecanediol (Sigma-Aldrich) into three-neck round-bottom flasks and a vacuum system was used to replace air with nitrogen. Second, 20 mL of diphenyl ether was mixed thoroughly, 4 mmol oleic acid and oleylamine were added, and the mixture was heated to 200 °C and maintained for 30 min. After the mixture cooled to room temperature, 2 volumes of ethanol were added to precipitate Fe_3_O_4_, and the solvent was removed by centrifugation at 6000 rpm for 3 min. Finally, the mixture was stored in hexane with 1 mmol of oleic acid and oleylamine.

For preparation of DPPC-Fe_3_O_4_, 2 mL of iron oxide-hexane solution was mixed with 18 mL of ethanol to precipitate the particles, and then a magnet was used to attract the iron oxide to remove the solvent and dry it under vacuum. Ten milligrams of nanoiron oxide powder was added to 5 µL of oleylamine and 500 µL of hexane. Then, 10 mg of DPPC (Avanti, Alabaster, AL, USA) was dissolved in 10 mL of ddH_2_O and mixed with iron oxide hexane solution, and ultrasonic vibration emulsification was performed for 30 min. Finally, the hexane was removed by heating and filtered with a 0.22 µm filter, and then magnetic filtration was used to concentrate and purify the DPPC-Fe_3_O_4_ nanoparticles.

### 2.6. Cell Culture and Gene Transfection

A mouse normal hepatocyte cell line, FL83B, was maintained in F-12K medium (Sigma-Aldrich) supplemented with 10% FBS (Gibco, Charlemont Terrace, Dublin, Ireland), 0.15% sodium bicarbonate (Sigma-Aldrich), 100 mM nonessential amino acids (NEAAs, Gibco), 100 unit/mL penicillin, and 100 µg/mL streptomycin (Gibco). Cultured cells were incubated at 37 °C with 5% CO_2_. Cells were seeded in a 6-well plate at a density of 5 × 10^5^ cells/well. After the cells grew to 80% confluence, the PmAlb-BDD-*FVIII*-pCMV-*EGFP* plasmid was transfected with Lipofectamine 2000 (Invitrogen) as described previously [37].

### 2.7. Physicochemical Analysis

Physicochemical analysis was performed by a Zetasizer analyzer (Malvern Instruments, London, UK). Dynamic light scattering (DLS) analysis of the nanoparticle size and distribution was performed, and the circular or linear form DNA was combined with DPPC-Fe_3_O_4_ nanoparticles and incubated 1 h at room temperature, then filled up to 1000 μL with ddH_2_O and transferred to cuvette for measurement at 25 °C. Each measurement was performed in triplicate. Electrophoretic light scattering (ELS) was used to measure the zeta potential of the nanoparticle. As described above, the different forms of DNA were combined with DPPC-Fe_3_O_4_ nanoparticles and incubated for 1 h at room temperature, then filled up to 997 μL with ddH_2_O and 3 μL of 1 M KCl was added and loaded into folded capillary cells for measurement. Each measurement was performed in triplicate.

### 2.8. Transmission Electron Microscopy (TEM)

TEM was performed to visualize the confirmations of circular and linearized plasmid DNA/DPPC-Fe_3_O_4_ nanoparticles in an aqueous environment as previously described [38]. Briefly, the plasmid DNA/DPPC-Fe_3_O_4_ complex was deposited on the carbon-coated copper grid. Then, it was stained with 2% uranyl acetate and analyzed by TEM (JEM-1400Flash TEM, JEOL Ltd., Tokyo, Japan).

### 2.9. Electrophoretic Gel Mobility Shift Assay (EMSA)

Electrophoretic mobility of DPPC-Fe_3_O_4_ carrier/DNA solutions was performed by loading the nanoparticle complexes into a 0.8% agarose gel containing SafeView™ dye (BioPioneer Tech., Taipei, Taiwan) and then quantified by ImageJ as previously described [39]. The percentage binding ability (%) = (band intensity (DNA alone)–band intensity (DNA binding))/band intensity (DNA alone) × 100%.

### 2.10. Quantitative Real-Time Reverse Transcription-PCR (qRT-PCR)

RNA was purified from the transfected cells using TRIzol^®^ Reagent (Invitrogen). cDNA was generated from 1 μg of purified RNA using the ImProm-II™ Reverse Transcription System (Roche, Basel, Kanton Basel-Stadt, Switzerland). Foreign gene expression was detected by qRT-PCR using *EGFP* and BDD-*FVIII* primer sets (Table 1), and *β-actin* mRNA was used as a loading control. qRT-PCR was applied in a Biometra T3000 thermocycler (Biometra, Westburg, The Netherlands) [40].

### 2.11. Gene Delivery via Tail Vein Injection

The PmAlb-BDD-*FVIII*-pCMV-*EGFP* plasmid (3 µg DNA/recipient) was mixed with DPPC-Fe_3_O_4_ and incubated for 1 h at room temperature. Then, Ringer’s solution was added to 200 µL, and mouse tail vein injection was performed (*n* = 5–7). The mice were held with a restrainer to carry out the tail vein injection, and the tail was immersed in warm water for 5 min. DPPC-Fe_3_O_4_-plasmid or Ringer’s solution was injected with a 27-gauge needle for 5–10 s with smooth buffer flow. After we confirmed that the tail did not bleed after pulling out the needle, the mice were returned to the cage.

### 2.12. Flow Cytometric Analysis

Cultured cells or homogenized liver tissues were trypsinized and fixed with fixing solution (1% FBS and 4% formaldehyde in PBS) for 10 min at 37 °C, permeabilized with 100% methanol for 30 min on ice, and blocked with 4% BSA in PBS for 1 h at room temperature. Then, human FVIII antibody (1:250 dilutions; Abcam, Cambridge, UK) was added overnight for incubation at 4 °C, and Alexa Fluor^®^ 546 dye-conjugated secondary antibody (1:500) was added at room temperature for 1 h. Samples were filtered and analyzed by flow cytometry (Accuri^®^ C6 Plus; BD Biosciences, Franklin Lakes, NJ, USA) [41].

### 2.13. Evaluation of Coagulation Phenotypic Restoration

The activated partial thromboplastin time (aPTT) test was executed to evaluate phenotypic coagulation restoration. Briefly, 1–3 weeks after injection of the DPPC-Fe_3_O_4_ plasmid complex, the mice were anesthetized with 1.4% isoflurane, and 90 µL of blood was collected from the retro-orbital sinus and mixed with 10 µL of 3.2% sodium citrate. Citrated blood was added to a Coag Dx Analyzer (IDEXX, Westbrook, ME, USA). When the measurement was done, the time was recorded.

### 2.14. Detection of Blood Biochemical Parameters

Biochemical parameters were used to confirm whether this therapy has side effects on the liver. Briefly, serum was prepared by adding blood into a Vacutainer (Becton Dickinson, BD, Franklin Lakes, NJ, USA), which was placed at room temperature for 30 min to allow clotting. After 30 min, the blood was centrifuged at 12,000 rpm for 2 min at room temperature, and the supernatant was collected to test the biochemical parameters. The liver biochemical parameters, including alanine aminotransferase (ALT), aspartate aminotransferase (AST), and alkaline phosphatase (ALKP), were detected by a VetTest Chemistry Analyzer (IDEXX, Westbrook, ME, USA).

### 2.15. Histologic Section Analysis

Liver tissue was collected after sacrifice and fixed in 10% formalin overnight. Then, the fixed liver tissue was dehydrated in a serial ethanol gradient (80%, 95%, 95%, and 100%) and xylene for 1 h. Finally, the liver tissue was embedded in paraffin and sliced on glass slides.

The DPPC-Fe_3_O_4_ distribution was detected by Prussian stain. Briefly, first, 10% ferric ferrocyanide and 20% HCl were used to stain liver tissue slides for 30 min, and the slides were washed twice with deionized water. Then, the cells were stained with nuclear fast red solution for 5 min and washed twice with deionized water. Finally, ferric and nuclear proteins were detected by ZEISS AXIO SCOPE.A1 microscope (ZEISS, Oberkochen, Germany).

### 2.16. Statistical Analysis

All data are presented as the mean ± SD and were compared using two-tailed unpaired Student’s *t* test. A value of *p* < 0.05 was considered statistically significant.

## 3. Results

### 3.1. Liver-Specific Promoter Activity in Different Types of Cell Lines

To test promoter activity in four different liver-specific promoter constructs, Pα1-AT, Eα1-AT, EPα1-AT, and PmAlb, we used seven different cell lines (Hepa1-6, MEF, C2C12, CHO, A549, Caco-2, and Ca922) to conduct the dual-luciferase reporter assays. The liver-specific promoters were linked to the luciferase (*Luc*) reporter gene and cloned into the pGL3-enhancer vector (Figure 1A). The fluorescence ratio of firefly/Renilla among these 7 cell lines is shown in Figure 1B. The PmAlb and Pα1-AT constructs exhibited higher promoter activities than the other two constructs (*p* < 0.01). Moreover, in Hepa1-6, a mouse liver cancer cell line, the PmAlb promoter construct had the highest fluorescence value compared with the Pα1-AT, Eα1-AT, and EPα1-AT promoter constructs, with 1.8-, 32.4-, and 21.3-fold increases, respectively (*p* < 0.001; Figure 1B). Therefore, the PmAlb promoter was chosen for further construction with human BDD-*FVIII* cDNA (Figure 1C) as a gene therapy vector.

### 3.2. Characterization of DPPC-Fe_3_O_4_ Carrying Different Forms of Plasmid DNA

**Figure 2:**
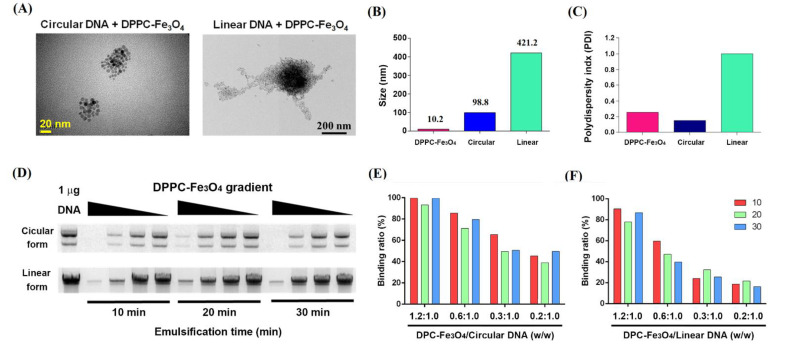
Characteristics of DPPC-Fe_3_O_4_ nanoparticle binding with circular or linear plasmid DNA. (**A**) Transmission electron microscopy (TEM) examination of the nanoparticle morphology of DPPC-Fe_3_O_4_ binding with circular plasmid DNA (right; scale bar = 20 nm) and linear plasmid DNA (left; scale bar = 200 nm). (**B**) Dynamic light scattering (DLS) analysis of the nanoparticle sizes of DPPC-Fe_3_O_4_ alone, DPPC-Fe_3_O_4_ binding with circular plasmid, and DPPC-Fe_3_O_4_ binding with linear plasmid DNA. (**C**) DLS analysis of the polydispersity index (PDI) value of DPPC-Fe_3_O_4_ alone, DPPC-Fe_3_O_4_ binding with circular plasmid, and DPPC-Fe_3_O_4_ binding with linear plasmid DNA. (**D**) Electrophoretic gel mobility shift assay (EMSA) of different emulsification times and concentrations of DPPC-Fe_3_O_4_ (1273.6 ng, 636.8 ng, 318.4 ng, and 212.3 ng) binding with 1000 ng circular or linear plasmids. The DPPC-Fe_3_O_4_/DNA ratios (*w*/*w*) were defined as 1.2:1.0, 0.6:1.0, 0.3:1.0, and 0.2:1.0, respectively. Semiquantitative analysis of the binding ability (×100%) of DPPC-Fe_3_O_4_ with circular plasmid DNA (**E**) or linear plasmid DNA (**F**).

To determine the morphology of DPPC-Fe_3_O_4_ after binding with either circular or linear plasmid DNAs, we performed transmission electron microscopy (TEM) imaging (Figure 2A). The results showed that DPPC-Fe_3_O_4_ to circular DNA exhibited a tighter conformation than DPPC-Fe_3_O_4_ bound to linear plasmid DNA. The particle size and size distribution of the DPPC-Fe_3_O_4_ plasmid complex were further analyzed by a Zetasizer analyzer. The data showed that the particle size of DPPC-Fe_3_O_4_ binding with circular plasmid DNA was 98.87 ± 3.64 nm, whereas the particle size of DPPC-Fe_3_O_4_ binding with linear plasmid DNA was 421.20 ± 23.96 nm (*p* < 0.01; Figure 2B). Furthermore, the polydispersity index (PDI) of DPPC-Fe_3_O_4_ binding with circular plasmid DNA was significantly lower than that of DPPC-Fe_3_O_4_ binding with linear plasmid DNA (0.15 vs. 1.0, *p* < 0.01; Figure 2C).

To investigate the binding ability of DPPC-Fe_3_O_4_ nanoparticles, we used different concentrations of DPPC-Fe_3_O_4_ bound with either circular or linear plasmid DNA under different emulsification times (10, 20, and 30 min) and performed EMSA gel electrophoresis (Figure 2D). Ideally, if DPPC-Fe_3_O_4_ and plasmid DNA are thoroughly combined, the unbound DNA band intensity will be weaker in the gel image. The results showed that a higher concentration and lower emulsification time of DPPC-Fe_3_O_4_ resulted in a higher binding ability with the same patterns of circular and linear plasmid DNAs (Figure 2D). Furthermore, the binding ability of DPPC-Fe_3_O_4_ nanoparticles combined with circular plasmid DNA (Figure 2E) was significantly better than that of linear plasmid DNA (Figure 2F). These results indicated that DPPC-Fe_3_O_4_ binding with circular plasmid DNA is more condensed and stable and thus suitable for use in gene therapy.

### 3.3. Characterization of Different Doses of DPPC-Fe_3_O_4_ with Circular Plasmid DNA

**Figure 3:**
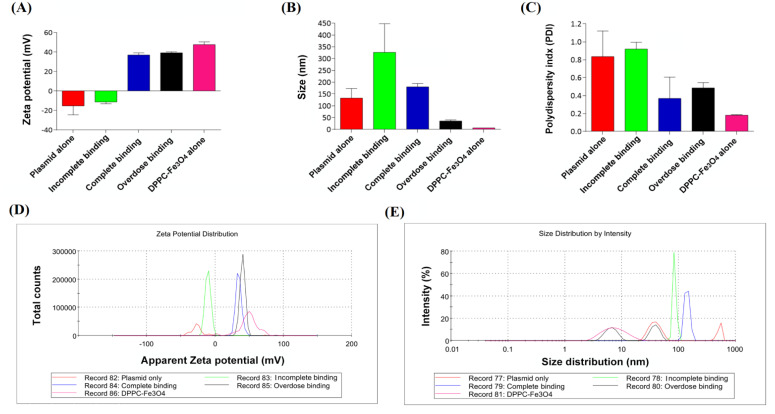
DLS analysis of nanoparticle characteristics in different dosages of DPPC-Fe_3_O_4_ binding with a constant amount of circular form plasmid DNA. (**A**) Average zeta potential (mV), (**B**) average size (nm), (**C**) polydispersity index (PDI) value, (**D**) profile of zeta potential distribution, and (**E**) profile of size distribution in different groups of circular plasmid DNA alone, DPPC-Fe_3_O_4_ alone, DPPC-Fe_3_O_4_-plasmid incomplete binding (200 ng DPPC-Fe_3_O_4_: 1000 ng circular DNA = 0.2:1.0; *w*/*w*), Fe_3_O_4_-plasmid complete binding (1000 ng DPPC-Fe_3_O_4_: 1000 ng circular DNA = 1.0:1.0; *w*/*w*), and Fe_3_O_4_-plasmid overdose binding (4000 ng DPPC-Fe_3_O_4_: 1000 ng circular DNA = 4.0:1.0; *w*/*w*). Data are presented as the mean ± SD (*n* = 3).

To determine the changes in zeta potential, particle size distribution, and PDI value in circular plasmid DNA, DPPC-Fe_3_O_4_ alone, and the DPPC-Fe_3_O_4_-plasmid complex, we performed Zetasizer Nano-ZS analyses (Figure 3A–E). The results showed that the zeta potential of nude circular plasmid DNA was −25.4 mV, which is a negative charge, and the zeta potential of the DPPC-Fe_3_O_4_ nanomaterial was +45.2 mV, which is a positive charge (Figure 3A). As the concentration of DPPC-Fe_3_O_4_ increased, the zeta potential of the DPPC-Fe_3_O_4_-plasmid DNA complex rose and changed from negative to positive: −10.4 mV for incomplete binding (DPPC-Fe_3_O_4_:circular DNA = 0.2:1.0; *w*/*w*), +34.6 mV for complete binding (DPPC-Fe_3_O_4_:circular DNA = 1.0:1.0), and +40.3 mV for overdose binding (DPPC-Fe_3_O_4_:circular DNA = 4.0:1.0) (Figure 3A,D). Furthermore, the results showed a gradient decrease in the particle size of the DPPC-Fe_3_O_4_-plasmid DNA complex from incomplete binding to overdose binding (Figure 3B), and the peak of the particle size distribution was more concentrated in the complete binding group (Figure 3E). Interestingly, the determined PDI index of the complete binding group also showed the lowest value among the three different binding conditions (Figure 3C). These results indicated that the status of completely bound nanoparticles was nearly monodisperse and was acceptable for delivery of DNA in vivo.

### 3.4. Evaluation of PmAlb-BDD-FVIII Circular Plasmid Expression in Mouse Hepatocytes

**Figure 4:**
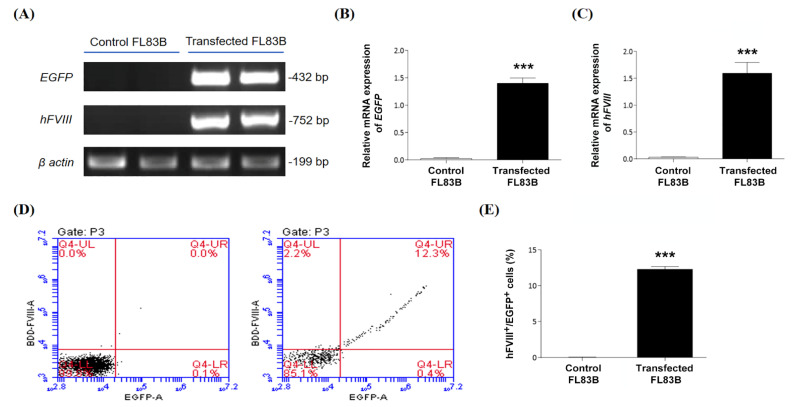
Assessment of PmAlb-*BDD-FVIII*-pCMV-*EGFP* plasmid DNA expression in normal mouse liver cells. (**A**) Representation of quantitative RT-PCR results of *EGFP* and *hFVIII* gene expression in FL83B cells after transfection with PmAlb-*BDD-FVIII*-pCMV-*EGFP* plasmid DNA for two days. Nontransfected FL83B cells were used as a negative control. A *β-actin* gene was used as an internal mRNA loading control. (**B**) Quantification of *EGFP* gene expression in FL83B cells transfected with (transfected group) or without (control group) PmAlb-BDD-*FVIII*-pCMV-*EGFP* plasmid DNA. (**C**) Quantification of human *FVIII* gene expression in FL83B cells transfected with (transfected group) or without (control group) PmAlb-BDD-FVIII-pCMV-EGFP plasmid DNA. (**D**) Flow cytometry analysis of EGFP and FVIII expression in FL83B cells after transfection of the PmAlb-*BDD-FVIII* plasmid DNA. Transfected cells were stained with anti-hFVIII antibody conjugated with Alexa Fluor^®^ 546 dye, and hFVIII and EGFP dual fluorescence-positive cells were analyzed by flow cytometry (right). Nontransfected FL83B cells were used as a negative control (left). (**E**) The percentage of both EGFP- and FVIII-positive cells in a total of 100,000 cells was quantified. Data are presented as the mean ± SD, *** *p* < 0.001 vs. the control group (two-tailed *t* test).

To confirm the PmAlb-BDD-*FVIII*-pCMV-*EGFP* dual gene expression in normal liver cells, we transfected the circular plasmid DNA construct into an FL83B mouse hepatocyte line using Lipofectamine and analyzed the cells by qRT-PCR and flow cytometry (Figure 4). Two days after transfection, the mRNA of hepatocytes was extracted for cDNA synthesis and qRT-PCR detection (Figure 4A). The results showed that both the exogenous human BDD-*FVIII* gene (Figure 4B) and *EGFP* reporter (Figure 4C) were highly expressed in the transfected cells, while there were no signals of these two exogenous genes in the untreated control cells (Figure 4A–C).

Furthermore, the transfected hepatocytes were stained with anti-hFVIII antibody conjugated with Alexa Fluor^®^ 546 dye, and hFVIII and EGFP dual fluorescence-positive cells were analyzed by flow cytometry (Figure 4D). The results showed that approximately 12.3 ± 0.4% of the FL83B cells expressed both BDD-hFVIII and EGFP proteins, while there were no hFVIII or EGFP signals in the untreated control cells (Figure 4E). Data suggested that the PmAlb-BDD-*FVIII*-pCMV-*EGFP* circular plasmid can efficiently express the BDD-hFVIII protein in normal liver cells and simultaneously coexpress EGFP for tracking successful gene delivery in the liver cells of mice.

### 3.5. Coagulation Phenotypic Correction by Nanoparticle Gene Delivery

**Figure 5:**
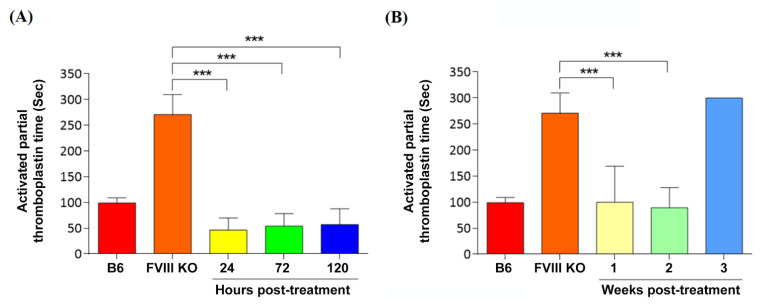
Assessment of coagulation restoration after DPPC-Fe_3_O_4_-PmAlb-*BDD-FVIII* gene therapy in mice with hemophilia A. (**A**) The aPTT test was performed at 24 h (*n* = 3), 72 h (*n* = 3), and 120 h (*n* = 4) after intravenous injection with the DPPC-Fe_3_O_4_-plasmid complex to evaluate the short-term therapeutic effect. Untreated FVIII knockout mice (*n* = 6) were used as a control for the disease group, and age-paired male C57BL/6J mice (*n* = 6) were used as a normal control group. (**B**) aPTT was tested weekly after intravenous injection with the DPPC-Fe_3_O_4_-plasmid complex to evaluate the long-term therapeutic effect. Data are presented as the mean ± SD, *** *p* < 0.001 vs. the untreated FVIII knockout mouse group (two-tailed *t* test).

To assess whether DPPC-Fe_3_O_4_ can deliver the PmAlb-BDD-*FVIII* plasmid and restore blood coagulation activity in a mouse model of hemophilia A, we performed an aPTT assay at different time points after injection of the DPPC-Fe_3_O_4_-plasmid complex (Figure 5). In short-term gene therapy assessment, the aPTT values of the DPPC-Fe_3_O_4_-plasmid complex recipients at 24-, 72-, and 120-h post-injection were 46.3 ± 23.5 s, 54.0 ± 24.4 s, and 57.0 ± 30.5 s, respectively, and all three groups exhibited significantly shorter blood coagulation times than the untreated *FVIII* knockout (*FVIII* KO) mice (270.7 ± 38.8 s, *n* = 7; *p* < 0.001; Figure 5A). In long-term gene therapy assessment, the aPTT values of the DPPC-Fe_3_O_4_-plasmid complex recipients at 1 week (100.1 ± 49.0 s; *n* = 5) and 2 weeks (89.3 ± 39.2 s; *n* = 5) post-injection exhibited normal blood coagulation function compared with that of the wild-type C57BL/6J (B6; 93.7 ± 22.5 s; *n* = 7) mice but did not maintain normal coagulation function after 3 weeks of DPPC-Fe_3_O_4_-plasmid complex injection (300.3 ± 20.4 s; Figure 5B). The data suggested that DPPC-Fe_3_O_4_-plasmid nanoparticles, as a novel gene therapy delivery system, can sustain the therapeutic effect for at least two weeks in *FVIII* KO hemophilic mice.

### 3.6. Distribution of DPPC-Fe_3_O_4_–Plasmid Nanoparticles in the Liver Tissue of Hemophilic Mice

**Figure 6:**
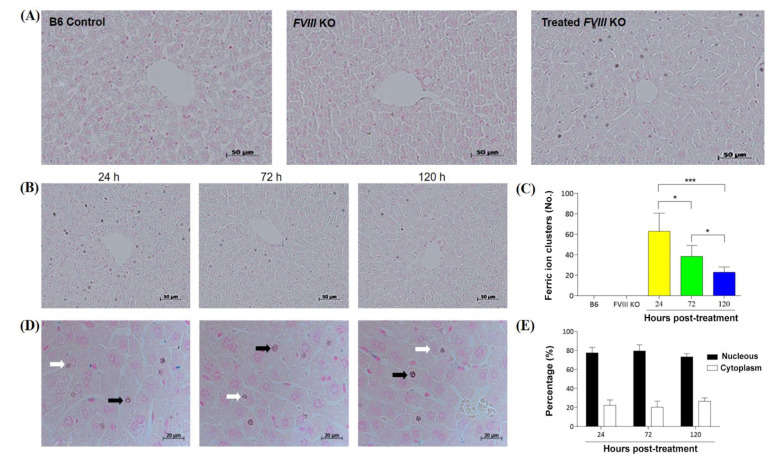
Perls’ Prussian blue staining to observe the localization of DPPC-Fe_3_O_4_-plasmid nanoparticles in the liver sections of recipient mice with hemophilia A. (**A**) Mice were sacrificed at 24 h (*n* = 3) after delivery of the DPPC-Fe_3_O_4_-plasmid complex, and Perls’ Prussian blue-stained iron oxide was observed in liver sections (right). C57BL/6J mice (*n* = 6) were used as a normal control (left). Untreated *FVIII* knockout mice (*n* = 6) were used as a negative control (middle). Images were obtained at 400 x magnification, scale bar = 50 µm. (**B**) Representative images of the Prussian blue-stained iron clusters at different time points. The recipient mice were sacrificed at 24 h (*n* = 3), 72 h (*n* = 3), and 120 h (*n* = 3) after delivery of the DPPC-Fe_3_O_4_-plasmid complex, and the liver sections were stained with Perls’ Prussian blue. Images were obtained at 400× magnification, scale bar = 50 µm. (**C**) Quantification of iron cluster numbers at different time points. (**D**) Representative images of the iron clusters in the cell nucleus (black arrows) or cytoplasm (white arrows). Images were obtained at 1000× magnification, scale bar = 20 µm. (**E**) Quantification of the percentage of iron clusters in the cell nucleus (black bar) or cytoplasm (white bar) at different time points by counting 100 clusters randomly. Data are presented as the mean ± SD, * *p* < 0.05, *** *p* < 0.001 (two-tailed *t* test).

To evaluate the distribution of DPPC-Fe_3_O_4_-plasmid nanoparticles in the recipient’s liver, we stained the liver tissue sections with Perls’ Prussian blue to detect the ferric ion of DPPC-Fe_3_O_4_ and the nucleus was stained with nuclear fast red solution (Figure 6). The results showed that blue-stained ferric ions could be easily detected in the liver tissue of the DPPC-Fe_3_O_4_-plasmid nanoparticle recipient mice, and most of the blue-stained ferric ion clusters were located around the hepatic sinusoid (Figure 6A). In addition, the DPPC-Fe_3_O_4_-plasmid complex levels decreased depending on the time post-treatment, as shown by analysis of the blue-stained ferric ion clusters. A higher number of blue-stained ferric ion clusters was present at 24 h post-treatment (63.0 ± 17.8 counts/view, *n* = 6), followed by 72 h post-treatment (38.5 ± 10.8 counts/view), and a lower number of clusters was shown at 120 h post-treatment (23.0 ± 5.2 counts/view); there were significant differences between each group (*p* < 0.05; Figure 6B,C). Furthermore, we investigated the percentage of clusters located in the nucleus or cytoplasm. We found 77.6 ± 5.7%, 79.7 ± 6.4%, and 73.3 ± 3.5% nuclear localization of blue-stained ferric ion clusters at 24 h, 72 h, and 120 h post-treatment, respectively (Figure 6D,E). The data indicated that most DPPC-Fe_3_O_4_-plasmid complexes can be efficiently transported into the nucleus of liver cells, which facilitates *hFVIII* gene expression in recipient mice with hemophilia A.

### 3.7. Safety Validation after Delivery of the DPPC-Fe_3_O_4_-Plasmid Complex

**Figure 7:**
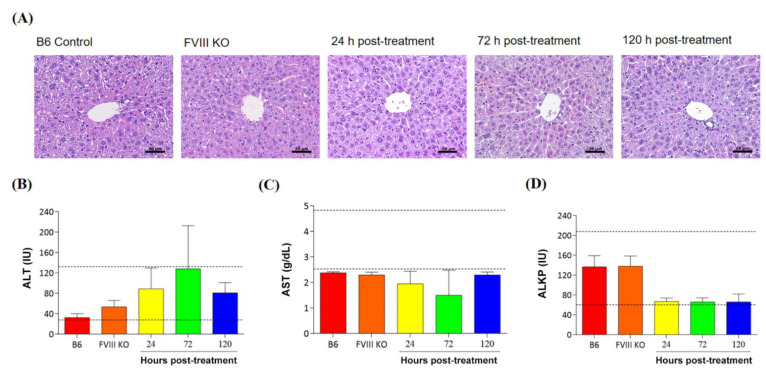
Verification of the side effects after delivery of the DPPC-Fe_3_O_4_-plasmid complex to the liver tissue of mice with hemophilia A. (**A**) The recipient mice were sacrificed at 24 h (*n* = 3), 72 h (*n* = 3), and 120 h (*n* = 3) after delivery of the DPPC-Fe_3_O_4_-plasmid complex, and the histopathological changes in H&E-stained liver sections were analyzed. C57BL/6 mice (*n* = 6) were used as a normal control, and *FVIII* knockout mice (*n* = 6) without DPPC-Fe_3_O_4_-plasmid complex injection were used as an untreated control. Images were obtained at 40× magnification, scale bar = 50 µm. Serum biochemical parameters for liver indices, including ALT (**B**), AST (**C**), and ALKP (**D**), were measured in the normal C57BL/6J control mice, *FVIII* knockout mice without treatment, and *FVIII* knockout mice treated with the DPPC-Fe_3_O_4_-plasmid complex at different time points (24 h, 72 h, and 120 h after gene therapy). The dashed lines show the normal range of each parameter in normal mice. Data are presented as the mean ± SD.

To confirm that DPPC-Fe_3_O_4_-plasmid complex therapy will not cause liver damage or side effects in recipient mice with hemophilia A, we performed blood biochemical parameter analyses and liver tissue H&E staining (Figure 7). The results showed that no pathological characteristics or inflammatory cell infiltration was found in the liver tissue sections of all groups (Figure 7A). In addition, blood biochemical parameters, including ALT, AST, and ALKP, were used to evaluate liver toxicity. The results showed that the average values of ALT (Figure 7B), AST (Figure 7C), and ALKP (Figure 7D) at all three time points (24 h, 72 h, and 120 h post-treatment) in the gene therapy groups and in the untreated *FVIII* KO and B6 groups were within the normal ranges of mouse blood biochemical parameters. The data suggested that the use of the novel DPPC-Fe_3_O_4_-plasmid nanoparticle for gene therapy is a safe approach in animals.

## 4. Discussion

In this study, three main findings were obtained. First, the mouse albumin (PmAlb) promoter expresses the highest degree of liver specificity and higher promoter activity than the Pα1-AT, Eα1-AT, and EPα1-AT promoter constructs. Second, the PmAlb-BDD-*FVIII*-pCMV-*EGFP* plasmid DNA can tightly bind to the DPPC-Fe_3_O_4_ nanomaterial, and the binding of circular plasmid is more efficient and compact than that of linear plasmid DNA. Third, the DPPC-Fe_3_O_4_-plasmid complex was easily detected in the liver of mice with hemophilia A after nanoparticle gene delivery by the tail vein, and the coagulation problem of these mice was restored for more than 2 weeks post-treatment.

In a dual-luciferase promoter assay, we identified a 1.06-kb length of PmAlb and a 581-bp length of Pα1-AT promoters that exhibited higher promoter activities among the four different promoter constructs in the Hepa1-6 liver cell line. However, two reconstructed liver-specific promoters, Eα1-AT and EPα1-AT, did not provide any advantage of promoter activity among seven different tissue-type cell lines in in vitro tests (Figure 1).

A previous study showed that gene therapy using a lentiviral vector targeted to hematopoietic stem cells or an AAV vector transcriptionally targeted to liver tissue is a feasible approach to treat mitochondrial neurogastrointestinal encephalomyopathy (MNGIE) [42]. Cabrera-Pérez et al. [43] further demonstrated that a human alpha-1-antitrypsin (hα1-AT) promoter improves the efficacy of an AAV vector for the gene therapy of MNGIE by regulating exogenous thymidine phosphorylase (TP) expression not only in the liver but also in the brain and small intestinal tissues. The same results were shown in our study: the Pα1-AT promoter-derived luciferase reporter was also highly expressed in C2C12 myoblast cells, CHO ovarian cells, Caco-2 intestinal cells, and MEF embryonic fibroblasts (Figure 1B). To avoid the possible side effects of recombinant BDD-*hFVIII* expression outside of liver tissue, such as induction of cerebral vascular infarction and pulmonary embolism, we chose the PmAlb promoter construct, which had highest liver-specific regulation, as a gene therapy backbone in this study.

Fe_3_O_4_ ionic nanoparticles have been developed as a common carrier in targeted drug delivery and are widely used to carry drugs for the treatment of diseases [33,44]. Furthermore, a lipid-coated nanoparticle, DPPC, has recently been developed as a nonviral delivery system to transport DNA or drugs into cells [28]. However, the mechanism of action and efficacy of the combination of DPPC with Fe_3_O_4_, as a novel drug delivery system, are still unclear. Therefore, in this study, we carefully assessed the binding efficiency of different forms of plasmid DNA, the DPPC-Fe_3_O_4_ concentration, and different emulsifying times by detecting the morphology, size, zeta potential, and PDI value. In TEM observations, we found that the DPPC-Fe_3_O_4_-PmAlb-BDD-*FVIII* circular plasmid DNA cluster was more compact and had a smaller particle size than the linear plasmid DNA combined with DPPC-Fe_3_O_4_ nanoparticles (Figure 2A) due to the loose structure of the primary form of linear DNA. In addition, we found that a 10 min emulsifying time is suitable for the preparation of DPPC-Fe_3_O_4_-PmAlb-BDD-*FVIII* nanoparticles when using EMSA gel mobility and quantitative analysis (Figure 2D). DPPC-Fe_3_O_4_-carrying DNA nanoparticles with a small size might completely combine the supercoiled circular plasmid DNA and can achieve a higher transfection efficiency in the targeted cells, possibly through membrane endocytic machinery. In a previous study, Lehner et al. [38] also demonstrated a similar result: a better transfection efficiency with polyethylenimine (PEI)-carrying circular form DNA nanoparticles compared to that of linear form DNA was found. Under electron microscopy observation, the researchers also found a remarkable difference in the shape of the DNA nanoparticle complexes: circular DNA had a smaller size with a well compacted and roughly spherical shape, while PEI-carried linear DNA nanoparticles appeared larger in size with string-like strand confirmation [38].

Currently, hemophilia A patients are treated with inconvenient and costly supplemental therapy involving FVIII protein with a short half-life via repeated intravenous injections with an interval of a couple days. Prolonged gene therapy by lipid-coated nanoparticle-encapsulated DNA or mRNA encoding human *FVIII* can solve this unmet medical need. A recent report showed that a single injection of BDD-*FVIII* mRNA-containing lipid nanoparticles at different dosages had a sufficient therapeutic efficacy [45]. However, the mRNA-lipid nanoparticle regimen rapidly decreased its therapeutic levels within 5–7 days post-treatment. In this study, we provided circular DNA-DPPC-Fe_3_O_4_ nanoparticle complexes that exhibit beneficial effects in extending the FVIII therapeutic efficacy duration up to 2–3 weeks in a mouse model of hemophilia A.

Traditionally, gene therapy is used to treat various inherited diseases mostly through virus-mediated gene delivery systems, such as lentiviral-, adenoviral-, or AAV-based vectors. However, there are some adverse effects in viral vector gene delivery approaches, including gene disruption, insertional mutagenesis, biotoxicity, and immunogenicity [46,47]. Moreover, the production of AAV vectors with genomes >4.7 kb is challenging because they frequently contain truncated genomes during viral packaging and are unsuitable for full-length FVIII cDNA (10.6 kb) or even B-domain deleted FVIII cDNA (4.35 kb) gene delivery [48]. A previous report also showed that some patients may generate neutralizing antibodies or endoplasmic reticulum stress, which influences the treatment effect of AAV-based gene delivery [27]. Therefore, lipid-coated nanoparticles, such as PEI and DPPC, have been developed as nonviral delivery systems to transport DNA or drugs into cells. In this study, our results suggest that DPPC-Fe_3_O_4_ nanoparticles can be a better material to conduct FVIII gene delivery because of their high biocompatibility, low biotoxicity, and lack of size limitation for carrying DNA. No pathological characteristics or inflammatory cell infiltration was found in the liver tissues, and all hepatic biochemical parameters, including ALT, AST, and ALKP, were within the normal ranges in recipient mice with hemophilia A after 3 weeks of DPPC-Fe_3_O_4_ nanoparticle treatment.

DPPC is a positively charged lipid-like nanoparticle that can tightly combine with negatively charged circular plasmid DNA, prevent therapeutic genes from degrading by circulating or intracellular enzymes, and even tolerate temperature changes. Moreover, magnetic DPPC-Fe_3_O_4_ nanoparticles provide an extra advantage in that they are easily guided to a specific position, such as the abdominal liver, by an ex vivo magnetic field during tail vein injection [49]. In addition, Prussian blue staining revealed ferrous ions in numerous nuclei in liver tissue, indicating that the DPPC-Fe_3_O_4_ plasmid complex can easily enter the liver cell nucleus after intravenous injection and facilitate exogenous *FVIII* gene expression.

FVIII inhibitor is a crucial issue for the treatment of hemophilia A. Patients with hemophilia might produce the FVIII antibody after repeatedly receiving the FVIII replacement therapy and then lose the therapeutic effect [22]. In FVIII gene therapy, it raises the same concern of inhibitor production; therefore, the *FVIII* gene can be modified to escape from immune system. A recent study showed that substitution of five amino acids in the A1 domain of human FVIII with the corresponding porcine FVIII residues could enhance secretion and gene therapy efficiency and did not exhibit an immunogenicity risk [50]. Moreover, the A2 and C2 domains of human FVIII have been confirmed to contain the immune response recognized sites [51,52]. Therefore, we speculated that replacement of these immune recognized residues of FVIII may avoid the production of inhibitory antibodies.

In this study, we successfully developed a novel DPPC-Fe_3_O_4_-plasmid complex to deliver the BDD-*hFVIII* gene for more than two weeks in a mouse model of hemophilia A. Four possible approaches to prolong nonviral gene therapy in future studies have been proposed. First, the amount of DNA might not be enough to maintain long-term existence in hepatic cells of recipient mice (3 µg plasmid DNA/recipient). In previous studies, we found that more than 10 µg of plasmid DNA has been used in different nonviral delivery systems, such as polyethylenimine, intravenous naked DNA injection, or human islet transplantation, to enhance and prolong the expression of foreign genes [53,54,55]. Hence, the plasmid DNA emulsified with DPPC-Fe_3_O_4_ nanoparticles can be increased 3–4-fold to improve the efficacy of this gene therapy. Second, a reduction in the DPPC-Fe_3_O_4_-plasmid DNA nanoparticle size can improve its cell membrane endocytosis efficiency [56,57] through modification of the flash nanocomplexation (FNC) preparation method [58], shrinking the DPPC-DNA nanoparticle size, to increase permeability in the liver cell and nucleus membranes. Third, the pH value is also an important factor that influences the net charge of nanoparticles and leads to poor condensation of the plasmid DNA [58]. Thus, we can adjust the pH values during DPPC-Fe_3_O_4_-plasmid complex production. Last, the size of the plasmid vector might be too large to transfect cells [59]. Therefore, a new minimal piggyback vector system [60] to remove the vector backbone of approximately 4 kb to generate mini-sized plasmid DNA will improve transfection efficiency and enhance foreign gene persistence in the liver of recipient mice.

## 5. Conclusions

In this study, we successfully developed a novel DPPC-Fe_3_O_4_-plasmid complex to deliver liver-specific albumin promoter-driven BDD-*hFVIII* gene expression for more than two weeks in a mouse model of hemophilia A. The binding ability of circular DNA was confirmed to be more stable than that of linear DNA when combined with DPPC-Fe_3_O_4_ nanoparticles. Furthermore, no liver toxicity, thromboses, deaths, or persistent changes were found in this DPPC-Fe_3_O_4_-plasmid gene therapy after safety validation. We concluded that this novel gene therapy can effectively improve abnormal hemostasis in *FVIII* KO mice and provide a possible strategy to treat patients with hemophilia A in clinical settings.

## Figures and Tables

**Table 1:** Information on the PCR or RT-PCR primers used in this study.

Gene Name	Primer Sequence (5′ to 3′)	Tm (°C)	Amplicon Size (bp)
*Pα1*-*AT*	F: 5′-ACCGAGGCACAGAGAGGTT-3′ R: 5′-GCCATTCACAAGGATACTGT-3′	67	581
*Eα1*-*AT*	F: 5′-CTTCAGCATCAGGCATTTTGG-3′ R: 5′-TCTCCAGAACCTCTCGCAGT-3′	63	473
*PmAlb*	F: 5′-TCACTCAAAAGAGTCCTGAA-3′ R: 5′-AGAAAGACTCGCTCTAATATAC-3′	57	1060
*EGFP*	F: 5′-GACTTCTTCAAGTCCGCCATGC-3′ R: 5′-CTCCAGCAGGACCATGTGAT-3′	55	432
*BDD*-*hFVIII*	F: 5′-CAGACTTTCGGAACAGAGGCA-3′ R: 5′-ATCTTTTTCCAGGTCAACATCA-3′	55	752
*β*-*actin*	F: 5′-CCGTCTTCCCCTCCATCGTGGG-3′ R: 5′-AGATCATTGTAGAAGGTGTGG-3′	68	199

## Data Availability

Not applicable.

## References

[1] Oldenburg J., Ananyeva N.M., Saenko E.L. (2004). Molecular basis of hemophilia A. Haemophilia.

[2] Goodeve A.C., Peake I.R. (2003). The molecular basis of hemophilia A: Genotype-phenotype relationships and inhibitor development. Semin. Thromb. Hemost..

[3] Ketterling R.P., Bottema C.D.K., Phillips J.A., Sommer S.S. (1991). Evidence that descendants of three founders constitute about 25% of hemophilia B in the United States. Genomics.

[4] Bolton-Maggs P.H., Pasi K.J. (2003). Haemophilias A and B. Lancet.

[5] Peyvandi F., Garagiola I., Young G. (2016). The past and future of hemophilia: Diagnosis, treatments, and its complications. Lancet.

[6] Tantawy A.A., Matter R.M., Hamed A.A., Shams El Din El Telbany M.A. (2010). Platelet microparticles in immune thrombocytopenic purpura in pediatrics. Pediatr. Hematol. Oncol..

[7] Gouw S.C., van der Bom J.G., Ljung R., Escuriola C., Cid A.R., Claeyssens-Donadel S., van Geet C., Kenet G., Mäkipernaa A., Molinari A.C. (2013). Factor VIII products and inhibitor development in severe hemophilia A. N. Engl. J. Med..

[8] Preissner K.T. (2008). Physiology of blood coagulation and fibrinolysis. Hamostaseologie.

[9] Gringeri A., Mantovani L.G., Scalone L., Mannucci P.M., COCIS Study Group (2003). Cost of care and quality of life for patients with hemophilia complicated by inhibitors: The COCIS Study Group. Blood.

[10] Mannucci P.M. (2003). Hemophilia: Treatment options in the twenty-first century. J. Thromb. Haemost..

[11] Mahlangu J., Oldenburg J., Paz-Priel I., Negrier C., Niggli M., Mancuso M.E., Schmitt C., Jiménez-Yuste V., Kempton C., Dhalluin C. (2018). Emicizumab prophylaxis in patients who have hemophilia A without inhibitors. N. Engl. J. Med..

[12] Oldenburg J., Mahlangu J.N., Kim B., Schmitt C., Callaghan M.U., Young G., Santagostino E., Kruse-Jarres R., Negrier C., Kessler C. (2017). Emicizumab prophylaxis in hemophilia A with inhibitors. N. Engl. J. Med..

[13] Astermark J., Donfield S.M., DiMichele D.M., Gringeri A., Gilbert S.A., Waters J., Berntorp E., FENOC Study Group (2007). A randomized comparison of bypassing agents in hemophilia complicated by an inhibitor: The FEIBA NovoSeven Comparative (FENOC) Study. Blood.

[14] Zhao Y., Weyand A.C., Shavit J.A. (2021). Novel treatments for hemophilia through rebalancing of the coagulation cascade. Pediatric Blood Cancer.

[15] Mikaelsson M., Oswaldsson U., Jankowski M.A. (2001). Measurement of factor VIII activity of B-domain deleted recombinant factor VIII. Semin. Hematol..

[16] Chen C.M., Wang C.H., Wu S.C., Lin C.C., Lin S.H., Cheng W.T.K. (2002). Temporal and spatial expression of biologically active human factor VIII in the milk of transgenic mice driven by mammary-specific bovine α-lactalbumin regulation sequences. Transgenic Res..

[17] Lusher J.M., Lee C.A., Kessler C.M., Bedrosian C.L. (2003). The safety and efficacy of B-domain deleted recombinant factor VIII concentrate in patients with severe hemophilia A. Haemophilia.

[18] Ren X., Gong X., Cai Q., Guo X., Xu M., Ren Z., Zeng Y. (2015). Efficient stabilization of recombinant human coagulation factor VIII in the milk of transgenic mice using hFVIII and vWF co-expression vector transduction. Biotechnol. Lett..

[19] Meeks S.L., Josephson C.D. (2006). Should hemophilia treaters switch to albumin-free recombinant factor VIII concentrates. Curr. Opin. Hematol..

[20] Parti R., Schoppmann A., Lee H., Yang L. (2005). Stability of lyophilized and reconstituted plasma/albumin-free recombinant human factor VIII (ADVATE RAHF-PFM). Haemophilia.

[21] Bardi E., Astermark J. (2015). Genetic risk factors for inhibitors in hemophilia A. Eur. J. Haematol..

[22] Astermark J. (2015). FVIII inhibitors: Pathogenesis and avoidance. Blood.

[23] VandenDriessche T., Thorrez L., Naldini L., Follenzi A., Moons L., Berneman Z., Collen D., Chuah M.K.L. (2002). Lentiviral vectors containing the human immunodeficiency virus type-1 central polypurine tract can efficiently transduce nondividing hepatocytes and antigen-presenting cells in vivo. Blood.

[24] Hacein-Bey-Abina S., von Kalle C., Schmidt M., Le Deist F., Wulffraat N., McIntyre E., Radford I., Villeval J.-L., Fraser C.C., Cavazzana-Calvo M. (2003). A serious adverse event after successful gene therapy for X-linked severe combined immunodeficiency. N. Engl. J. Med..

[25] Kattenhorn L.M., Tipper C.H., Stoica L., Geraghty D.S., Wright T.L., Clark K.R., Wadsworth S.C. (2016). Adeno-associated virus gene therapy for liver disease. Hum. Gene Ther..

[26] Pasi K.J., Rangarajan S., Mitchell N., Lester W., Symington E., Madan B., Laffan M., Russell C.B., Li M., Pierce G.F. (2020). Multiyear follow-up of AAV5-hFVIII-SQ gene therapy for hemophilia A. N. Engl. J. Med..

[27] Fong S., Handyside B., Sihn C.R., Liu S., Zhang L., Xie L., Murphy R., Galicia N., Yates B., Minto W.C. (2020). Induction of ER stress response by an AAV5 BDD FVIII construct is dependent on the strength of the hepatic-specific promoter. Mol. Ther. Methods Clin. Dev..

[28] Cristofolini L., Berzina T., Erokhina S., Konovalov O., Erokhin V. (2007). Structural study of the DNA dipalmitoylphosphatidyl-choline complex at the air−water interface. Biomacromolecules.

[29] Filion M.C., Phillips N.C. (1997). Toxicity and immunomodulatory activity of liposomal vectors formulated with cationic lipids toward immune effector cells. Biochim. Biophys. Acta.

[30] Laurent S., Forge D., Port M., Roch A., Robic C., Vander Elst L., Muller R.N. (2008). Magnetic iron oxide nanoparticles: Synthesis, stabilization, vectorization, physicochemical characterizations, and biological applications. Chem. Rev..

[31] Singh N., Jenkins G.J.S., Asadi R., Doak S.H. (2010). Potential toxicity of superparamagnetic iron oxide nanoparticles (SPION). Nano Rev..

[32] Ling D., Hyeon T. (2013). Chemical design of biocompatible iron oxide nanoparticles for medical applications. Small.

[33] Stefaniu C., Brezesinski G., Möhwald H. (2012). Polymer-capped magnetite nanoparticles change the 2D structure of DPPC model membranes. Soft Matter.

[34] Wang J., Chen Y., Chen B., Ding J., Xia G., Gao C., Cheng J., Jin N., Zhou Y., Li X. (2010). Pharmacokinetic parameters and tissue distribution of magnetic Fe_3_O_4_ nanoparticles in mice. Int. J. Nanomed..

[35] Ou-Yang H., Wu S.C., Sung L.Y., Yang S.H., Yang S.H., Chong K.Y., Chen C.M. (2021). STAT3 is an upstream regulator of *granzyme G* in the maternal-to-zygotic transition of mouse embryos. Int. J. Mol. Sci..

[36] Yen C.C., Chang W.H., Tung M.C., Chen H.L., Liu H.C., Liao C.H., Lan Y.W., Chong K.Y., Yang S.H., Chen C.M. (2020). Lactoferrin protects hyperoxia-induced lung and kidney systemic inflammation in an in vivo imaging model of NF-κB/luciferase transgenic mice. Mol. Imaging Biol..

[37] Tsai S.W., Wu H.S., Chen I.A., Chen H.L., Chang G.R., Fan H.C., Chen C.M. (2019). Recombinant porcine myostatin propeptide generated by the *Pichia pastoris* elevates myoblast growth and ameliorates high-fat diet-induced glucose intolerance. Res. Vet. Sci..

[38] Lehnera R., Wanga X., Hunziker P. (2013). Plasmid linearization changes shape and efficiency of transfection complexes. Eur. J. Nanomed..

[39] Hsu C.Y.M., Uludağ H. (2008). Effects of size and topology of DNA molecules on intracellular delivery with non-viral gene carriers. BMC Biotechnol..

[40] Lan Y.W., Yang J.C., Yen C.C., Huang T.T., Chen Y.C., Chen H.L., Chong K.Y., Chen C.M. (2019). Predifferentiated amniotic fluid mesenchymal stem cells enhance lung alveolar epithelium regeneration and reverse elastase-induced pulmonary emphysema. Stem Cell Res. Ther..

[41] Tsou Y.A., Chang W.C., Lin C.D., Chang R.L., Tsai M.H., Shih L.C., Staniczek T., Wu T.F., Hsu H.Y., Chang W.D. (2021). Metformin increases survival in hypopharyngeal cancer patients with diabetes mellitus: Retrospective cohort study and cell-based analysis. Pharmaceuticals.

[42] Torres-Torronteras J., Gomez A., Eixarch H., Palenzuela L., Pizzorno G., Hirano M., Andreu A.L., Barquinero J., Martí R. (2011). Hematopoietic gene therapy restores thymidine phosphorylase activity in a cell culture and a murine model of MNGIE. Gene Ther..

[43] Cabrera-Pérez R., Vila-Julià F., Hirano M., Mingozzi F., Torres-Torronteras J., Martí R. (2019). Alpha-1-antitrypsin promoter improves the efficacy of an adeno-associated virus vector for the treatment of mitochondrial neurogastrointestinal encephalomyopathy. Hum. Gene Ther..

[44] Xu Z., Hao C., Xie B., Sun R. (2019). Effect of Fe_3_O_4_ nanoparticles on mixed POPC/DPPC monolayers at air-water interface. Scanning.

[45] Chen C.Y., Tran D.M., Cavedon A., Cai X., Rajendran R., Lyle M.J., Martini P.G.V., Miao C.H. (2020). Treatment of hemophilia A using Factor VIII messenger RNA lipid nanoparticles. Mol. Ther. Nucleic Acids.

[46] Loring H.S., ElMallah M.K., Flotte T.R. (2016). Development of RAAV2-CFTR: History of the first rAAV vector product to be used in humans. Hum. Gene Ther. Methods.

[47] Thomas C.E., Ehrhardt A., Kay M.A. (2003). Progress and problems with the use of viral vectors for gene therapy. Nat. Rev. Genet..

[48] Nambiar B., Cornell Sookdeo C., Berthelette P., Jackson R., Piraino S., Burnham B., Nass S., Souza D., O’Riordan C.R., Vincent K.A. (2017). Characteristics of minimally oversized adeno-associated virus vectors encoding human factor VIII generated using producer cell lines and triple transfection. Hum. Gene Ther. Methods.

[49] Israel L.L., Galstyan A., Holler E., Ljubimova J.Y. (2020). Magnetic iron oxide nanoparticles for imaging, Targeting and treatment of primary and metastatic tumors of the brain. J. Control. Release.

[50] Cao W., Dong B., Horling F., Firrman J.A., Lengler J., Klugmann M., de la Rosa M., Wu W., Wang Q., Wei H. (2020). Minimal essential human factor VIII alterations enhance secretion and gene therapy efficiency. Mol. Ther. Methods Clin. Dev..

[51] Wuerth M.E., Cragerud R.K., Spiegel P.C. (2015). Structure of the human factor VIII C2 domain in complex with the 3E6 inhibitory antibody. Sci. Rep..

[52] Markovitz R.C., Healey J.F., Parker E.T., Meeks S.L., Lollar P. (2013). The diversity of the immune response to the A2 domain of human factor VIII. Blood.

[53] Shimoda M., Chen S., Noguchi H., Matsumoto S., Grayburn P.A. (2010). In vivo non-viral gene delivery of human vascular endothelial growth factor improves revascularisation and restoration of *euglycaemia* after human islet transplantation into mouse liver. Diabetologia.

[54] Boletta A., Benigni A., Lutz J., Remuzzi G., Soria M.R., Monaco L. (1997). Nonviral gene delivery to the rat kidney with polyethylenimine. Hum. Gene Ther..

[55] Liu F., Huang L. (2002). Noninvasive gene delivery to the liver by mechanical massage. Hepatology.

[56] Inoh Y., Nagai M., Matsushita K., Nakanishi M., Furuno T. (2017). Gene transfection efficiency into dendritic cells is influenced by the size of cationic liposomes/DNA complexes. Eur. J. Pharm. Sci..

[57] Betzer O., Shilo M., Opochinsky R., Barnoy E., Motiei M., Okun E., Yadid G., Popovtzer R. (2017). The effect of nanoparticle size on the ability to cross the blood–brain barrier: An in vivo study. Nanomedicine.

[58] Santos J.L., Ren Y., Vandermark J., Archang M.M., Williford J.-M., Liu H.-W., Lee J., Wang T.-H., Mao H.-Q. (2016). Continuous production of discrete plasmid DNA-polycation nanoparticles using flash nanocomplexation. Small.

[59] Yin W., Xiang P., Li Q. (2005). Investigations of the effect of DNA size in transient transfection assay using dual luciferase system. Anal. Biochem..

[60] Troyanovsky B., Bitko V., Pastukh V., Fouty B., Solodushko V. (2016). The functionality of minimal piggyBac transposons in mammalian cells. Mol. Ther. Nucleic Acids.

